# Contribution of Auditory Working Memory to Speech Understanding in Mandarin-Speaking Cochlear Implant Users

**DOI:** 10.1371/journal.pone.0099096

**Published:** 2014-06-12

**Authors:** Duoduo Tao, Rui Deng, Ye Jiang, John J. Galvin, Qian-Jie Fu, Bing Chen

**Affiliations:** 1 Department of Otology and Skull Base Surgery, Eye Ear Nose and Throat Hospital, Fudan University, Shanghai, China; 2 Division of Communication and Auditory Neuroscience, House Research Institute, Los Angeles, California, United States of America; 3 Department of Head and Neck Surgery, David Geffen School of Medicine, UCLA, Los Angeles, California, United States of America; The University of Chicago, United States of America

## Abstract

**Purpose:**

To investigate how auditory working memory relates to speech perception performance by Mandarin-speaking cochlear implant (CI) users.

**Method:**

Auditory working memory and speech perception was measured in Mandarin-speaking CI and normal-hearing (NH) participants. Working memory capacity was measured using forward digit span and backward digit span; working memory efficiency was measured using articulation rate. Speech perception was assessed with: (a) word-in-sentence recognition in quiet, (b) word-in-sentence recognition in speech-shaped steady noise at +5 dB signal-to-noise ratio, (c) Chinese disyllable recognition in quiet, (d) Chinese lexical tone recognition in quiet. Self-reported school rank was also collected regarding performance in schoolwork.

**Results:**

There was large inter-subject variability in auditory working memory and speech performance for CI participants. Working memory and speech performance were significantly poorer for CI than for NH participants. All three working memory measures were strongly correlated with each other for both CI and NH participants. Partial correlation analyses were performed on the CI data while controlling for demographic variables. Working memory efficiency was significantly correlated only with sentence recognition in quiet when working memory capacity was partialled out. Working memory capacity was correlated with disyllable recognition and school rank when efficiency was partialled out. There was no correlation between working memory and lexical tone recognition in the present CI participants.

**Conclusions:**

Mandarin-speaking CI users experience significant deficits in auditory working memory and speech performance compared with NH listeners. The present data suggest that auditory working memory may contribute to CI users' difficulties in speech understanding. The present pattern of results with Mandarin-speaking CI users is consistent with previous auditory working memory studies with English-speaking CI users, suggesting that the lexical importance of voice pitch cues (albeit poorly coded by the CI) did not influence the relationship between working memory and speech perception.

## Introduction

The cochlear implant (CI) has been very successful in restoring hearing and communication to many adult and pediatric patients with severe hearing loss. Despite this success, CI speech performance remains much poorer than that of normal hearing (NH) listeners, and there is much variability in CI outcomes [1]–[6]. Previous studies have used demographic information (e.g., age at implantation, duration of deafness, etc.), etiology of deafness, CI device type and speech processing strategy, educational and family background to explain the variability in CI outcomes, but with limited success [7], [8]. Other factors may also contribute to the variability in CI outcomes, such as CI users' perceptual, cognitive and linguistic capabilities. Pisoni and colleagues [9]–[11] have investigated some of these “higher-level” measures to explain individual differences in information processing that may underlie speech performance and language development.

One such higher-level process is working memory, which can be defined as a temporary storage mechanism for awareness, sensory perception or information retrieved from long-term memory [12]. Short-term memory may be considered to be a subset of working memory. In speech, short-term working memory is used to encode, store, maintain, and retrieve phonological and lexical representations of words for both speech perception and production [13]. Short-term working memory bridges the sensory input and a listener's long-term pattern representations. One way to assess short-term working memory is to measure the number of familiar items that can be recalled in correct serial order. Digit span recall is commonly used to measure verbal working memory capacity. Forward digit span requires a listener to repeat a sequence of digits in the correct order; backward digit span requires a listener to repeat a sequence of digits in reverse order [9], [10], [14]–[18]. Forward digit span is considered to be a measure of rapid phonological coding skill, with relatively little cognitive processing demand, while backward digit span requires greater cognitive load [19], [20]. Forward and backward digit span can be used to estimate the capacity of short-term working memory [10].

Verbal rehearsal speed (i.e., articulation rate) can be used to measure the efficiency of short-term working memory [10]. Articulation rate is measured by asking listeners to repeat words in meaningful or non-meaningful sentences, which typically contains approximately seven syllables [21]. Different from digit span tasks, articulation rate may reflect listeners' ability to keep and retrieve information in short-term memory for more complex linguistic processes, such as word recognition, sentence recognition and speech production. Articulation rate, a measure of working memory efficiency, has been correlated with working memory capacity [9], [10], [22], [23].

Previous studies have shown that short-term working memory is linked to NH children's ability to recognize and learn new words [24], [25]. NH children's ability to mimic the sound of nonsense words has also been correlated with vocabulary and novel word learning [24], [26]. Other research [27] has suggested that speech and language development are closely associated with verbal working memory. Speech perception and language processing are closely correlated, and both depend on rapid and efficient phonological coding of speech in short-term memory [13], [25], [28].

When the auditory periphery is impaired, listeners may experience a greater cognitive load that may be due to limited capacity and/or efficiency of short-term working memory. Stiles et al. [29] compared performance of six- to nine-year-olds with mild to moderate hearing loss to that of NH peers for phonological and visuospatial tasks that targeted short-term memory. Although articulation rate and vocabulary were lower in hearing-impaired (HI) children than in NH peers, there was no significant difference in speech performance between NH and HI children. This result is in contrast to those for children who use a CI. Watson et al [30] found significantly lower digit span and articulation rate (using nonsense words) performance for CI children than for NH peers. Pisoni et al. [22] reported similar results for digit span performance in older CI children. Watson et al [30] compared cortical recordings and working memory performance in age-matched NH and CI listeners. Results showed a significant correlation between the mismatch negativity activation (pre-attentive) and auditory working memory in NH children, but not in CI children. The authors suggested that severe hearing impairment may have disrupted or limited language development in CI children. Taken together, these studies also suggest that severe hearing loss, as experienced by pediatric CI users, may also impair normal development of short-term working memory.

Pisoni and Geers [9] found significant correlations between auditory digit span and speech performance in 43 pediatric CI participants with a relatively homogeneous demographic background, even after several years of CI experience [22]. Pisoni and Cleary [10] measured forward and backward digit span, articulation rate and spoken word recognition in 176 pediatric CI participants. Correlation analyses showed that 20% of the variance in word recognition scores could be explained by articulation rate (i.e., working memory efficiency), with 7% of the variance explained by digit span performance (i.e., working memory capacity). Nittrouer et al. [31] measured serial recall of non-rhyming nouns, rhyming nouns and non-rhyming adjectives in NH adults and children, as well as CI children. Results showed that CI children were less accurate in serial recall than NH children. However, the rate of recall did not significantly differ between CI and NH children, suggesting that working memory capacity, rather than working memory efficiency (as suggested by the Pisoni studies), may explain the variance in speech performance among CI children.

Given these somewhat inconsistent findings regarding the effects of short-term working memory on CI users' speech performance, we compared working memory and speech in young Mandarin-speaking CI listeners. In this study, we investigated the relationship between working memory and understanding of lexical tones (single syllables), disyllables, and sentences in Mandarin-speaking listeners. We hypothesized that, consistent with previous studies with English-speaking listeners, Mandarin-speaking CI listeners would exhibit deficits in both working memory capacity and efficiency, compared with NH listeners. We also hypothesized that the different linguistic processing demands of a tonal language such as Mandarin Chinese might affect the relationship between working memory and speech performance, compared to previous studies with English-speaking listeners. Similar to previous studies by Pisoni and colleagues, working memory capacity was assessed using forward and backward digit span recall, and working memory efficiency was estimated using articulation rate.

## Materials and Methods

### Ethics Statement

This study was approved by the Institutional Review Board protocol of Shanghai Eye Ear Nose and Throat Hospital, Fudan University, China. Written informed consent was obtained from each participant prior to enrollment in this study.

### Participants

Thirty-two CI users (21 pre-lingually deafened and 11 post-lingually deafened) participated in the experiment and completed all tests. All CI subjects were unilaterally implanted. CI participants all used the same device (Nucleus-24; Cochlear Corp.) and speech-processing strategy (Advanced Combination Encoder, or ACE). CI participants' mean age at testing was 13.0 years (SD = 4.0, range: 6.0–26.0), age at implantation was 7.7 years (SD = 5.2, range: 2.0–25.0) and mean experience with device was 5.2 years (SD = 2.3, range: 0.3–11.0). Detailed demographic information is listed in Table 1. A control group of 21 NH listeners also participated in the experiment and completed all tests. All NH participants passed hearing screening at 20 dB HL from 250 to 8000 Hz in both ears. NH participants' mean age was 11.0 years (SD = 1.6, range: 8–14). Although the sample size was different between the two subject groups, a one-way Kruskal-Wallis analysis of variance (ANOVA) on ranked data showed no significant difference in age between groups (p = 0.161). Similarly a Fisher exact test showed no significant difference in the gender distribution between groups (p = 0.159).

**Table 1:** Demographic information of all CI participants.

Participant	Age (yrs)	Sex	Age at Implantation(yrs)	CI experience (yrs)	Pre/Post-lingually deafened	Hearing Aid Experience (yrs)	Duration of Deafness (yrs)
S1	15	F	4	11	Pre	0	15
S2	10	M	2	8	Pre	0	9
S3	6	F	2	4	Pre	0	5
S4	19	M	12	7	Pre	4	18
S5	11	M	3	8	Pre	0	10
S6	11	F	6	5	Pre	0	10
S7	8	F	4	4	Pre	0	7
S8	13	M	9	4	Pre	9	12
S9	9	F	4	5	Pre	2	9
S10	9	M	4	5	Pre	0	7
S11	7	F	5	2	Pre	3	6
S12	13	M	2	11	Pre	0	12
S13	9	M	4	5	Pre	0	6
S14	11	F	2	9	Pre	0	10
S15	13	M	2	11	Pre	0	12
S16	16	M	12	4	Pre	0	16
S17	11	M	2	9	Pre	0	10
S18	10	M	2	8	Pre	0	10
S19	10	M	4	6	Pre	0	9
S20	8	M	3	5	Pre	0	7
S21	8	F	3	5	Pre	2	7
S22	12	M	7	5	Post	1	8
S23	18	F	17	1	Post	7	2
S24	13	M	10	3	Post	0	4
S25	16	M	11	5	Post	8	13
S26	23	M	22	1	Post	21	21
S27	23	F	23	0.3	Post	11	11
S28	26	M	25	1	Post	0	24
S29	9	F	7	2	Post	0	4
S30	11	F	7	4	Post	0	8
S31	23	F	20	3	Post	8	17
S32	14	M	8	6	Post	0	9

Note: Yrs  =  years; F  =  female; M  =  male; CI  =  cochlear implant.

CI participants were tested while wearing their clinically assigned speech processors and settings; once set, they were asked to not change these settings during the course of testing. As shown in Table 1, 11 CI subjects wore hearing aids (HAs) during their everyday listening experience. During testing, the HA was removed, and these subjects were tested using the CI only. The contralateral acoustic hearing ear was not plugged during testing.

### Test methods and materials

Assessment measures included a broad range of auditory and memory tasks. Working memory capacity was measured using forward digit span and backward digit span; working memory efficiency was measured using articulation rate. Speech perception was assessed with: (a) word-in-sentence recognition in quiet, (b) word-in-sentence recognition in noise, (c) Chinese disyllable recognition in quiet, (d) Chinese lexical tone recognition in quiet. Self-reported data were also collected regarding participants' performance in schoolwork. All testing was performed in a sound treated listening booth; participants were seated 1 m away from a single loudspeaker. All stimuli were presented at 65 dBA.

#### Auditory digit span

Forward and backward auditory digit span recall was measured using an adaptive (1-up/1-down) procedure. Stimuli included digits zero through nine produced by a single male talker. During testing, digits were randomly selected and presented in sequence in an auditory-only context (no visual cues). Participants responded by clicking on the response boxes (labeled “0” through “9”) shown on a computer screen that in order of the sequence that they heard. The initial sequence contained three digits. Depending on the correctness of response, the number of digits presented was either increased or decreased (the sequence was adjusted by two digits for the first two reversals and by one digit size for the subsequent reversals). Each test run contained 25 trials. The digit span score represented the mean number of digits that could be correctly recalled averaged across all but the first two reversals. For the forward digit span test, participants were asked to recall the sequence of digits in the order presented. For the backward digit span test, participants were asked to recall the sequence of digits in reverse order from the original presentation.

#### Sentence recognition

Recognition of words in sentences in quiet and in noise was assessed using the Mandarin Speech Perception (MSP) materials, which consists of 10 lists of 10 sentences each, produced by a single female talker [32]. Each sentence contains seven monosyllabic words, resulting in a total of 70 monosyllabic words for each list. For testing in noise, steady noise was spectrally shaped to match the average spectrum across all sentences produced by the female talker. The signal-to-noise ratio (SNR) was fixed at +5 dB. During testing a sentence list was randomly selected, and a sentence from that list was randomly selected and presented to the participant, who repeated the sentence as accurately as possible. Subjects were instructed that each sentence contained seven words, and to guess at words they did not understand. If the participant gave no response or only a partial response, the tester repeated the sentence and tried to elicit a more complete repetition of all seven words in the sentence. Performance was scored in terms of the percentage of words in sentences correctly identified; two lists were tested for each participant, and scores were averaged across the two runs.

#### Articulation rate

Articulation rate was estimated from participant responses recorded during the assessment of word-in-sentence recognition in quiet. For each participant, the mean duration of the repetition of all seven syllables was used to calculate the articulation rate, similar to [10]. Participants were instructed that each sentence would contain seven syllables, and to guess at the syllables if they were unsure. Articulation rate measures were obtained for all sentence repetitions, whether or not they were repeated correctly.

#### Disyllable recognition

Mandarin disyllables, like spondees in English, consist of two stressed syllables, each of which contains a lexical tone. Disyllables are most widely used in daily life by Chinese Mandarin-speaking people. Disyllable recognition was assessed using the Mandarin Disyllable Recognition Test (DRT), which consists of 10 lists of 35 disyllables each [33]. The disyllabic test lists were phonemically balanced in three dimensions: vowels, consonants and Chinese tones. During testing, a test list was randomly selected and stimuli were randomly selected from within the list (without replacement) and presented to the participant, who repeated the disyllable as accurately as possible. The tester calculated the percentage of syllables correctly identified in disyllabic words. All syllables in the DRT were scored, resulting in a total of 70 monosyllabic words for each list. No trial-by-trial feedback was provided during the test. Two of the 10 lists were randomly selected and used to test each participant.

#### Mandarin lexical tone recognition

Lexical tone recognition was measured for four tonal patterns: Tone 1 (flat fundamental frequency, or F0), Tone 2 (rising F0), Tone 3 (falling-rising F0), and Tone 4 (falling F0). Stimuli were taken from the Standard Chinese Database [34]. Sixteen Mandarin Chinese words (/ba/,/bo/,/bi/,/bu/ in Pinyin) were produced by two male and two female talkers, resulting in a total of 64 tokens. In each trial, a tone was randomly selected from the stimulus set and presented to the listener. Participants responded by clicking on one of four choices shown on a computer screen, labeled as “Tone 1”, “Tone 2,” “Tone 3”, and “Tone 4”. The mean percent correct was calculated across two runs for each participant. No training or trial-by-trial feedback was provided.

#### Self-reported school rank

Participants (or their parents) were asked to rate their performance in schoolwork. A five-point visual analog scale was used to obtain ratings, with 1 = 0–20% rank in class, 2 = 21–40% rank in class, 3 = 41–60% rank in class, 4 = 61–80% rank in class, and 5 = 81–100% rank in class.

## Results

**Figure 1:**
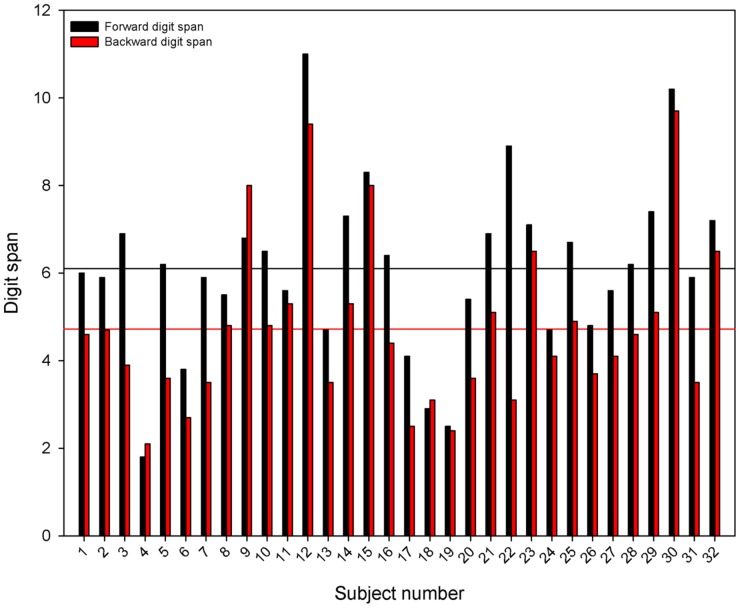
Forward and backward digit span scores for individual CI participants. The black bars show forward digit span data and the red bars show backward digit span data. The horizontal black line shows the mean forward digit span score and the horizontal red line shows the mean backward digit span score. CI  =  cochlear implant.

Figure 1 shows individual CI performance for forward (black bars) and backward digit span (red bars). Inter-subject variability was quite large, with performance ranging from 1.8 to 11 for forward digit span and from 2.1 to 9.7 for backward digit span.

**Figure 2:**
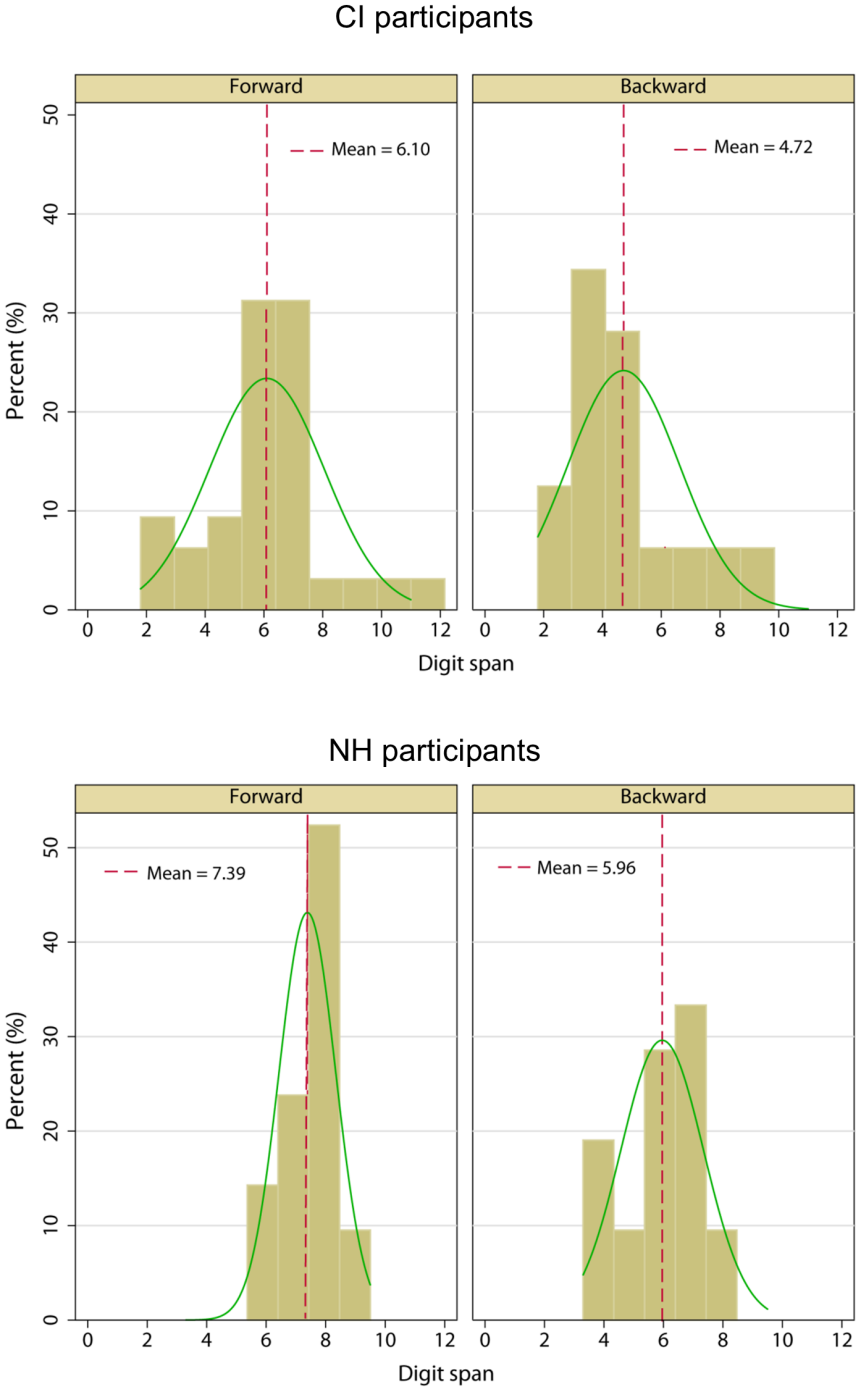
Distributions of forward and backward digit span scores for CI and NH participants. The left panels show forward digit span data and the right panels show backward digit span data. The top panels show CI data and the bottom panels show NH data. The vertical dashed lines show overall mean scores. The green lines show normal-density curve lines.

Figure 2 shows the distribution of scores for forward digit span (left panels) and backward digit span (right panels) for CI (top panels) and NH participants (bottom panels). The mean CI score was 4.72 (SE = 0.33) for backward digit span and 6.10 (SE = 0.35) for forward digit span. The distribution of forward digit span scores was not significantly different from the normal distribution (p = 0.382). However, the distribution of backward digit span scores was significantly different from normal (p = 0.019). The mean NH score was 5.96 (SE = 0.30) for backward digit span and 7.39 (SE = 0.21) for forward digit span. The distribution of span scores was not significantly different from the normal distribution for both forward (p = 0.495) and backward digit span (p = 0.236). A split-plot repeated measures analysis of variance (RM ANOVA) with digit span (forward or backward) as the within-subject factor and group (CI or NH) as the between-subject factor was performed on the CI and NH digit span data. Results showed that forward digit span scores were significantly better than backward digit span scores [F(1,51)  = 72.81, p<0.001] and that NH performance was significantly better than CI performance [F(1,51)  = 8.14, p = 0.006]; there were no significant interactions [F(1,51)  = 0.027, p = 0.871].

**Figure 3:**
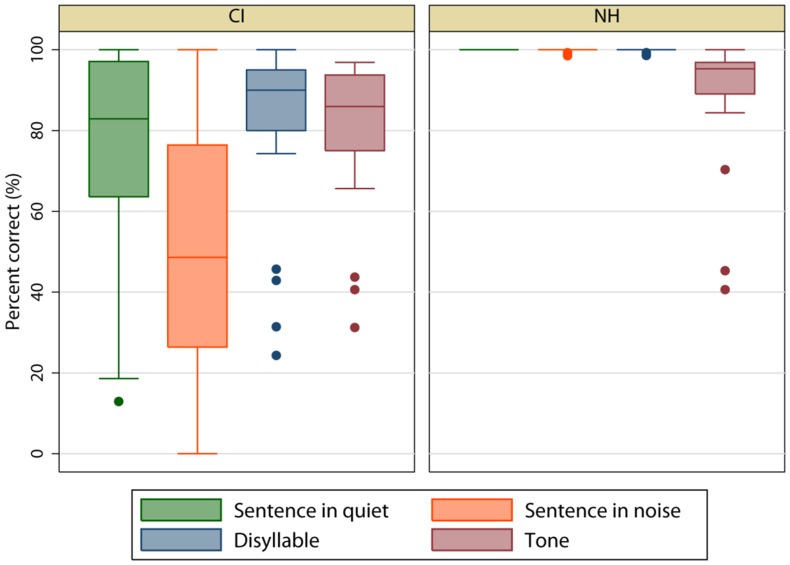
Boxplots of speech performance. The left panel shows CI data and the right panel shows NH data. Within each box, the horizontal line shows the mean, the error bars show the 10^th^ and 90^th^ percentiles, and the filled circles show outliers.

Figure 3 shows speech performance for CI (left panel) and NH participants (right panel). The mean CI percent correct was 77.43 (SE = 4.11) for sentence recognition in quiet, 49.68 (SE = 5.38) for sentence recognition in noise, 82.94 (SE = 3.43) for disyllable recognition, and 80.96 (SE = 2.94) for lexical tone recognition. The mean NH percent correct was 100 (SE = 0.00) for sentence recognition in quiet, 99.83 (SE = 0.10) for sentence recognition in noise, 99.7 (SE = 0.12) for disyllable recognition, and 88.39 (SE = 3.60) for lexical tone recognition.

As shown in Table 1, 11 of the 32 CI subjects used a contralateral HA for everyday listening. The HA was removed during testing, but unfortunately the HA ear was not plugged; also no audiometric data was available for the acoustic hearing ear. A two-way ANOVA was performed on all the CI data, with everyday hearing status (CI-only or CI+HA) and speech test (sentence recognition in quiet, sentence recognition in noise, disyllable recognition, lexical tone recognition, forward digit span, backward digit span and articulation rate) as factors. Results showed a significant effect of test [F(6,210)  = 179.188, p<0.001] but not for everyday hearing status [F(1,210)  = 1.237, p = 0.267]; there were no significant interactions [F(6,210)  = 1.407, p - 0.213]; Thus, while acoustic hearing without the HA may have been available to these subjects, there was no significant difference in performance between subjects who use only a CI in everyday listening and those who used a CI + HA.

A split-plot RM ANOVA with speech test (sentence recognition in quiet, sentence recognition in noise, disyllable recognition, and lexical tone recognition) as the within-subject factor and group (CI or NH) as the between-subject factor was performed on the CI and NH data. Results showed that significant effects for speech test [F(1,153)  = 19.03, p<0.001] and subject group [F(1,51)  = 1502.66, p<0.001]; there was a significant interactions [F(3,153)  = 30.35, p<0.001]. Because there was a significant interaction, within-subject effects were tested independently for each group. For CI participants, a one-way RM ANOVA on ranked data showed a significant effect of speech test (Chi-square  =  45.56 with 3 degrees of freedom, p<0.001). Tukey pair-wise comparisons showed that performance for sentence recognition in quiet, disyllable recognition, and lexical tone recognition were all significantly better than for sentence recognition in noise (p<0.05). For NH participants, a one-way RM ANOVA on ranked data showed a significant effect of speech test (Chi-square  =  49.40 with 3 degrees of freedom, p<0.001). Tukey pair-wise comparisons showed that performance for sentence recognition in quiet, sentence recognition in noise, and disyllable recognition were all significantly better than for lexical tone recognition (p<0.05). One-way ANOVAs on ranked data showed that NH performance was significant better than CI performance for all speech tests (p<0.05 in all cases).

Table 2 shows simple bivariate correlations between demographic factors and speech measures. Age at testing, duration of deafness, and age at implantation were significantly correlated with sentence recognition in quiet and in noise, as well as with disyllable recognition. Duration of deafness was also significantly correlated with tone recognition. None of the demographic factors were significantly correlated with self-reported school rank.

**Table 2:** Bivariate correlation between demographic variables and speech performance scores.

	SIQ	SIN	Disyllable	Tone	School rank
Age test	−0.56**	−0.63**	−0.65**	−0.39	−0.23
CI exp	0.31	0.41	0.44	0.19	0.10
Dur deaf	−0.55**	−0.51*	−0.57**	−0.47*	−0.40
Pre/post	−0.12	−0.26	−0.23	0.01	0.16
Age implant	−0.56**	−0.66**	−0.69**	−0.38	−0.22

r values are shown for each correlation. Age test  =  age at testing; CI exp  =  amount of experience with cochlear implant; Pre/Post  =  pre- or post-lingually deafened; Age implant  =  age at implantation; SIQ  =  sentence recognition in quiet; SIN  =  sentence recognition in noise.

*p **≤**0.01.

** *p* **≤**0.001.

**Figure 4:**
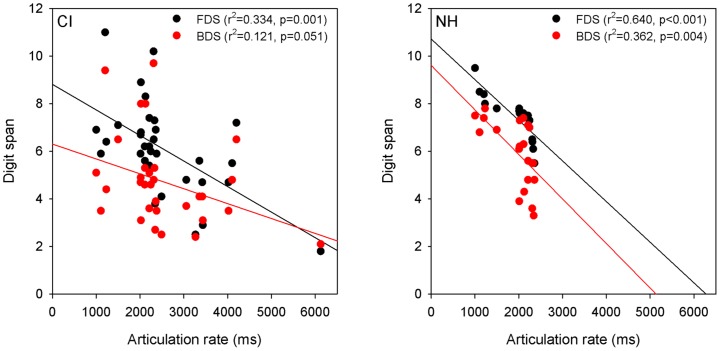
Forward and backward digit span as a function of articulation rate. The left panel shows CI data and the right panel shows NH data. The black circles show forward digit span data and the red circles show backward digit data. R squared values and *p* values are shown in the right of each panel. FDS  =  forward digit span; BDS  =  backward digit span.

Figure 4 shows forward (black circles) and backward (red circles) digit span as a function of articulation rate for CI (left panel) and NH participants (right panel). The mean CI articulation rate was 2526.1 ms (SE = 186.7), and the mean NH rate was 1951.2 ms (SE = 91.6). Because of the large variability in articulation rate values, especially for CI participants, articulation rate values were transformed to z-scores; the z-cores were used for subsequent analyses. Correlation analyses showed that CI participants' forward digit span was significantly correlated with articulation rate (r = −0.578, p = 0.001); the correlation between backward digit span and articulation rate failed to achieve significance (r = −0.348, p = 0.051). For NH participants, correlation analyses showed that articulation rate z-scores were significantly correlated with both forward (r = −0.800, p<0.001) and backward digit span (r = −0.602, p = 0.004).

**Figure 5:**
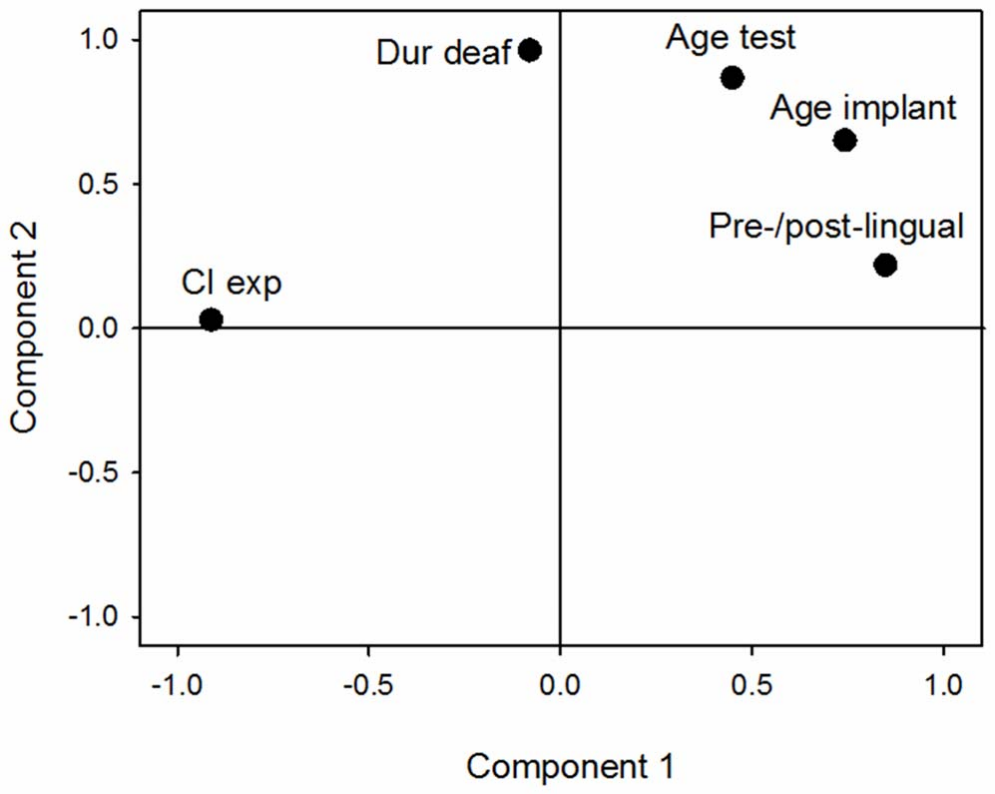
Factor loadings of demographic variables on two components. A varimax method was used to rotate the space. Age implant  =  age at implantation; Age test  =  age at testing; Dur deaf  =  duration of deafness; CI exp  =  CI experience; Pre-/post-lingual  =  pre- or post-lingually deafened.

To better understand the relationship between working memory and speech performance, it is important to control for demographic variables likely to contribute to speech performance. As shown in Table 2, age at testing, duration of deafness, and age at implantation were significantly correlated with most speech measures. Because these demographic factors may be inter-related, a factor analysis was performed to reduce the demographic data. Factor extraction was performed using principal components analysis (PCA) for the following demographic factors: age at testing, age at implantation, duration of deafness, CI experience, and pre- or post-lingually deafened. Table 3 shows the correlations among five demographic variables. Figure 5 shows the factor loadings relating each demographic variable to each factor plotted in varimax rotated space. Because there were two components (factors), two PCA factor scores were used in the later correlation analyses between working memory and speech tests. Given a threshold of 0.7 for factor loadings, the data in Figure 5 suggest that CI experience, age at implantation, and pre- or post-lingual deafness were strongly represented by Component 1, and that age at testing and duration of deafness were strongly represented by Component 2.

**Table 3:** Correlation coefficents matrix from Principle Component Analysis (PCA).

	Age implant	Dur deaf	CI exp	Pre/Post
Age test	0.91	0.75	−0.33	0.59
Age implant		0.56	−0.69	0.71
Dur deaf			0.03	0.11
CI exp				−0.59

Age test  =  age at testing; Age implant  =  age at implantation; Dur deaf  =  duration of deafness, CI exp  =  amount of experience with cochlear implant; Pre/Post  =  pre- or post-lingually deafened;

Table 4 shows simple bivariate correlations and partial correlations between speech measures and working memory performance. The effects of demographic factors were partialled out using the PCA data in the partial correlations. For the simple bivariate correlation analyses, forward digit span and articulation rate were significantly correlated with sentence recognition in quiet, disyllable recognition, and tone recognition; backward digit span was correlated only with disyllable recognition. For the partial correlation analyses, most of the significant bivariate correlations persisted when statistically controlling for demographic variables, except that articulation rate was no longer significantly correlated with sentence recognition in noise. Interestingly, after controlling for demographic variables, partial correlations showed that backward digit span was significantly correlated with sentence recognition in quiet (the simple bivariate correlation was not significant). For the simple bivariate correlations, forward digit span, backward digit span, and articulation rate were significantly correlated with self-reported school rank. After partialling out demographic variables, only forward and backward digit span remained significantly correlated with school rank.

**Table 4:** Correlations between working memory and speech performance scores.

	Simple Bivariate Correlations			Partial Correlations		
	FDS	BDS	AR	FDS	BDS	AR
SIQ	0.70**	0.43	−0.79**	0.70**	0.54*	−0.77**
SIN	0.43	0.18	−0.50*	0.42	0.28	−0.41
Disyllable	0.71**	0.49*	−0.62**	0.80**	0.72**	−0.58**
Tone	0.65**	0.41	−0.54*	0.60**	0.43	−0.46*
SR	0.69**	0.63**	−0.46*	0.64**	0.64**	−0.40

r values are shown for each correlation. For the partial correlations, the reduced data from the PCA were used to statistically control for demographic variables. SIQ  =  sentence recognition in quiet; SIN  =  sentence recognition in noise; SR  =  self-reported school rank; FDS  =  forward digit span; BDS  =  backward digit span; AR  =  articulation rate.

*p **≤**0.01.

** *p* **≤**0.001.

Forward digit span was significantly correlated with backward digit span for both CI (r  =  0.796; p<0.001) and NH participants (r = 0.647; p = 0.002). Table 5 shows correlations between working memory and speech performance while partialling out demographic factors using PCA data (as in Table 4) and partialling out either working memory capacity (forward and backward digit span) or efficiency (articulation rate). Because forward and backward digit span were significantly correlated, PCA data from factor analysis was used to reduce the working memory capacity data. When working memory capacity was partialled out, working memory efficiency was significantly correlated only with sentence recognition in quiet (r = −0.69; p<0.001). When working memory efficiency was partialled out, working memory capacity was significantly correlated only with disyllable recognition in quiet (r = 0.48; p = 0.006); working memory capacity was also significantly correlated with school rank (r = 0.61; p<0.001).

**Table 5:** Partial correlations between working memory and speech performance scores.

	WM efficiency	WM capacity
	(partial out WM capacity)	(partial out WM efficiency)
SIQ	−0.69**	0.40
SIN	−0.30	0.10
Disyllable	−0.37	0.48*
Tone	−0.28	0.40
SR	−0.13	0.61**

Working memory efficiency was represented by articulation rate data. Working memory capacity was represented by combined forward and digit span data, using PCA data from factor analysis. The reduced data from the PCA were used to statistically control for demographic variables. Each column shows r values when one of the working memory measures was partialled out. WM  =  working memory; SIQ  =  sentence recognition in quiet; SIN  =  sentence recognition in noise; SR  =  self-reported school rank.

*p **≤**0.01.

** *p* **≤**0.001.

## Discussion

Speech performance was significantly poorer for CI users than for NH listeners, for all speech measures. Mean CI subjects' Mandarin tone recognition, disyllable recognition and sentence recognition in quiet were all fairly good, better than 80% correct. However, sentence recognition in quiet was the most variable, with scores ranging from 12.9% to 100% correct. CI performance was poorest for sentence recognition in noise. Consistent with previous studies [9], [10], [22], [35], forward and backward digit span scores were significantly poorer for CI than for NH participants, although there was some overlap in the distributions of digit span scores. Articulation rate was also significantly longer for CI than for NH participants. Taken together, these measures of auditory working memory suggest that CI users may experience poorer and less efficient auditory information processing than NH listeners [23]. These findings support our hypothesis that Mandarin-speaking CI participants may exhibit deficits in phonological information-processing capacity and efficiency compared with NH participants that would be reflected in the present digit span and articulation rate tasks.

CI users' sentence recognition in noise was not significantly correlated with working memory measures (see Table 4). In contrast, articulation rate was significantly correlated with sentence recognition in quiet after controlling for demographic variables. The lack of correlation between sentence recognition in noise and working memory measures may have been due to masking of syllables in the sentences. As such, the items could not be reliably stored in the short-term working memory. Digit span recall and articulation rate were measured in quiet, where no such masking was present. The +5dB SNR proved very difficult for many of the present CI subjects, but has been used in previous CI studies. In Friesen et al. [36] study, sentence recognition in noise was tested at 0, +5, +10, and +15 dB SNR in adult CI users and NH listeners listening to acoustic CI simulations. With 8 channels, NH subjects scored ∼60% correct while CI users scored only ∼25% correct. Perhaps a better approach would have been to use an adaptive procedure to measure the speech reception threshold (SRT) - i.e., 50% correct words in sentences. Alternatively, a wider range of SNRs could have been tested.

Mean NH performance with disyllables and sentences in quiet or noise was nearly perfect, while mean performance with lexical tones was nearly 11 points poorer. Compared with the contextual cues available for sentence and disyllable recognition, the monosyllable words used to test tone recognition contained fewer contextual cues. Also, intelligibility among the four lexical tones is not evenly distributed. Lee and Lee [37], testing NH Mandarin-speaking musicians, found that Tone 1 was most easily identified, followed by Tone 3, Tone 4, and Tone 2. Tones 2 and 3 were most easily confused, with some confusion between Tones 1 and 4. Confusion among the present tone stimuli may have similarly reduced performance relative to the context-heavy sentence and disyllable stimuli.

Forward and backward digit span scores were significantly correlated in both CI and NH participants, consistent with previous studies [9], [10], [22], [38], [39]. Because digit span data scales positively (better performance with more digits recalled) and articulation rate scales negatively (better performance with lower rate), correlations between digit span and articulation rate were negative, also consistent with previous CI studies [9], [10], [22], [35], [39]. In a study by Pisoni and colleagues [25], mean articulation rate for adolescent English-speaking CI users was 2002 ms. Interestingly, this was a follow-up study for articulation rate data collected in the same subjects 8 years earlier. At the initial measure, articulation rates were higher (mean  =  3720 ms), suggesting that these CI users improved their working memory efficiency as they developed and gained experience with their device. In a recent study by Geers et al. [38], the mean articulation rate was 2024 ms for teenage CI users and 1777 ms for NH participants. In the present study, the articulation rate was slower for CI participants (mean  =  2526 ms, SE  =  186.7) than for NH participants (mean  =  1951 ms, SE = 91.6). Some differences in methodology between this study and the Pisoni and Geers studies may explain the slower articulation rates found here. In the Pisoni studies, subjects were provided with some visual cues (e.g., lip-reading and/or the text of the sentence), which most likely improved understanding before repeating the sentence. In the present study, no visual cues or feedback was provided, which may have made speech understanding more difficult and the articulation rate slower. In the Geers study, the mean CI subject age was 16.7 years and the mean CI experience was 13.3 years. In the present study the mean CI subject age was 13.0 years and the mean CI experience 5.2 years, which may have also contributed to the slower articulation rate.

The correlation analyses shown in Table 4 showed significant correlations between many working memory and speech measures, with some exceptions (e.g., forward and backward digit span versus speech in noise; backward digit span versus tone recognition). The subsequent analyses in Table 5 showed the relationship between working memory capacity, working memory efficiency, and speech performance. After controlling for demographic factors and partialling out working memory capacity (forward and backward digit span), working memory efficiency (articulation rate) was significantly correlated only with sentence recognition in quiet (Table 5). Working memory efficiency explained 48% of the variance in sentence recognition in quiet. Similarly, after partialling out working memory efficiency, working memory capacity was significantly correlated with disyllable recognition, also measured in quiet (Table 5). However, working memory capacity only explained 23% of the variance in disyllable recognition. Thus, the contribution of working memory capacity or efficiency seems to depend on the speech measure. For sentence recognition in quiet, the present findings are largely in agreement with Pisoni and colleagues [10], who argued that working memory efficiency explained a significant portion of the variability in CI speech performance. The results with disyllable recognition are more in agreement with Nittrouer et al [31], who argued that working memory capacity contributed to the variability in pediatric CI speech performance.

We hypothesized that the pattern of results might be different between Mandarin- and English-speaking populations, given the importance of F0 cues to tonal languages such as Mandarin Chinese. However, the main findings from the present study were consistent with previous studies in English-speaking participant populations. The correlation analyses in Table 4 showed significant correlations between tone recognition and forward digit span, as well as articulation rate. However, after partialling out either working memory capacity or efficiency, there was no significant correlation between tone recognition and working memory (Table 5). This finding is similar to that of Pisoni and Cleary [10], who found no significant correlation between monosyllable word recognition and working memory capacity after partialling out working memory efficiency. Different from our hypothesis, the present data suggest that the relationship between speech performance and working memory may not be affected by perception of F0 cues important to tonal languages such as Mandarin Chinese.

In this study, self-reported school rank was used to estimate participants' learning abilities. After controlling for demographic variables, working memory capacity explained 37% of the variance in CI participants' self-reported school rank when working memory efficiency was partialled out, consistent with findings by Alloway et al. [40]. However, school rank was not significant correlated with working memory efficiency when capacity was partialled out. Limitations to working memory can vary widely among NH children and is closely associated with children's learning ability [41], [42], and deficits in working memory have been associated with learning difficulties [28], [40], [43]–[44]. When the acoustic input is degraded as in the CI case, it is unclear how the reduced working memory may interact with CI users' learning capabilities. The present data suggest that learning difficulties for young Mandarin-speaking CI users may be associated with limited working memory capacity instead of efficiency.

## Conclusions

We compared auditory working memory measures with speech performance and in 32 Mandarin-speaking CI users and 21 NH participants. Major findings include:

1. Working memory performance was significantly poorer for CI than for NH participants, suggesting that Mandarin-speaking CI users experience limited working memory capacity (as measured with forward and backward digit span) and efficiency (as measured with articulation rate).

2. After controlling for demographic factors, CI users' forward digit span was significantly correlated with sentence recognition in quiet, disyllable recognition, tone recognition and school rank. Backward digit span was significantly correlated with sentence recognition in quiet, disyllable recognition, and school rank. Articulation rate was significantly correlated with sentence recognition in quiet, disyllable recognition, and tone recognition. Sentence recognition in noise was not significantly correlated with any working memory measure, possibly due the relatively low SNR used for testing.

3. After controlling for demographic factors and partialling out working memory capacity, working memory efficiency was significantly correlated only with sentence recognition in quiet. After partialling out working memory efficiency, working memory capacity was significantly correlated with disyllable recognition and school rank. This suggests that the contribution of working memory capacity and efficiency may depend on the speech measure.

4. The importance of F0 cues for tonal languages such as Mandarin Chinese did not appear to influence the relationship between working memory and speech understanding observed in previous studies with English-speaking listeners.
